# Lipedema stage affects adipocyte hypertrophy, subcutaneous adipose tissue inflammation and interstitial fibrosis

**DOI:** 10.3389/fimmu.2023.1223264

**Published:** 2023-07-28

**Authors:** Philipp Kruppa, Sabrina Gohlke, Kamila Łapiński, Francisco Garcia-Carrizo, George A. Soultoukis, Manfred Infanger, Tim J. Schulz, Mojtaba Ghods

**Affiliations:** ^1^ Department of Plastic, Aesthetic and Reconstructive Microsurgery/Hand Surgery, Hospital Ernst von Bergmann, Potsdam, Germany; ^2^ Department of Adipocyte Development and Nutrition, German Institute of Human Nutrition (DIfE) Potsdam-Rehbrücke, Nuthetal, Germany; ^3^ Otto-von-Guericke University Magdeburg, Department of Plastic, Aesthetic and Hand Surgery, Magdeburg, Germany; ^4^ German Center for Diabetes Research (DZD), München, Germany; ^5^ Institute of Nutritional Science, University of Potsdam, Nuthetal, Germany

**Keywords:** lipedema, lipoedema, fibrosis, adipose tissue, inflammation, macrophages

## Abstract

**Introduction:**

Lipedema is a painful subcutaneous adipose tissue (SAT) disease characterized by adipocyte hypertrophy, immune cell recruitment, and fibrosis in the affected areas. These features are thought to contribute to the development and progression of the condition. However, the relationship between lipedema disease stage and the associated adipose tissue changes has not been determined so far.

**Methods:**

SAT biopsies of 32 lipedema patients, ranging across the pathological stages I to III, and 14 BMI- and age-matched controls were harvested from lipedema-affected thighs and non-symptomatic lower abdominal regions. Histological and immunohistochemical (IHC) staining and expression analysis of markers for adipogenesis, immunomodulation, and fibrosis were performed on the tissue biopsies.

**Results:**

Lipedema patients showed increased adipocyte areas and a stage-dependent shift towards larger cell sizes in the thighs. Lipedema SAT was linked with increased interstitial collagen accumulation in the thighs, but not the lower abdominal region when compared to controls. There was a trend toward progressive SAT fibrosis of the affected thighs with increasing lipedema stage. Elevated gene expression levels of macrophage markers were found for thigh SAT biopsies, but not in the abdominal region. IHC staining of lipedema thigh biopsies confirmed a transiently elevated macrophage polarization towards an M2-like (anti-inflammatory) phenotype.

**Conclusions:**

In summary, lipedema SAT is associated with stage-dependent adipocyte hypertrophy, stage-progressive interstitial fibrosis and elevated proportion of M2-like macrophages. The character of the inflammatory response differs from primary obesity and may possess an essential role in the development of lipedema.

## 1 Introduction

Lipedema is considered a hormone-related adipose tissue disorder which predominantly affects women and is associated with severe pain. Based on the limited data available, epidemiological estimates indicate an approximate prevalence of 10% in the overall female population. The disease is characterized by a bilaterally symmetrical and disproportional expansion of the subcutaneous adipose tissue (SAT) of the extremities in relation to the torso (1). Conservative therapies, including manual lymphatic drainage, compression therapy, decongestive sports/movement therapy, skin care, and empowerment are currently considered as first line treatments (2–4). Surgical reduction of pathological SAT with lymph-sparing liposuction in wet-technique is another potential therapeutic option to improve symptoms, mobility and overall quality of life (5, 6). However, the disease is frequently left undiagnosed and receiving appropriate medical care remains a challenge for patients. To develop appropriate therapies for lipedema, the separation of the disease from possible differential diagnoses, such as obesity or lymphedema, is of particular importance. To date, the etiology and pathogenesis of lipedema have not been adequately clarified. Therefore, a reliable diagnosis “lipedema” is a significant limitation.

Recent studies have highlighted characteristic abnormalities for lipedema-affected adipose tissue, including dysfunctional blood and lymphatic vessels (microangiopathy) (7), excess interstitial fluid (7, 8), pathological increases in adipocyte size (hypertrophy) and/or proliferation (hyperplasia) (7, 9–12), and recruitment of inflammatory immune cells (7, 9, 13–15). It was shown that the increased immune cell activation was associated with macrophage infiltration (7, 9, 13, 15), while gene expression markers in lipedema biopsies indicate an elevated macrophage polarization towards an M2-like (anti-inflammatory) phenotype (13). These structural changes in connective tissue result in SAT remodeling with accumulation of collagen fibers as a sign of interstitial fibrosis (11, 16). The clinical classification for lipedema reflects this immune-modulated fibrotic remodeling and is based on palpation examination of the skin and subcutaneous tissue into three stages (**Table 1**). The palpable changes correspond to a gradual enlargement of the nodular tissue structures and increasing induration of the skin and subcutaneous tissue (17). However, it remains unclear to what extent the molecular and morphological changes of the lipedema SAT correspond to the clinically diagnosed lipedema stage.

**Table 1:** Stages of lipedema according to Strößenreuther [17].

	Skin	Adipose tissue	Texture/Palpation
Stage I	- Smooth skin texture - Soft	- Thickened (excess tissue) - Soft	- Small nodules (rice-like”/”pearl-sized”/”Styrofoam balls in a plastic bag”)
Stage II	- Irregular and uneven appearance with skin depressions - Hardened - Possibly inflamed	- Thickened (excess tissue) - Hardened (fibrotic changes)	- Nodules increase in size (“walnut-sized” to “apple-like”) - “Mattress pattern” indentations
Stage III	- Thickened and hardened (*peau d’orange*) - Loss in elasticity - Induration possible	- Thickened (excess tissue) - Hardened (fibrotic changes) - Large tissue extrusion causing deformations	- Numerous large and hardened subdermal nodules - Constant and palpable fibrosis

The currently used clinical classification for lipedema reflects an immune-modulated fibrotic remodeling and is based on palpation examination of the skin and subcutaneous tissue into three stages. The palpable changes correspond to a gradual enlargement of the nodular tissue structures and increasing induration of the skin and subcutaneous tissue.

Current literature considers lipedema-associated symptoms as largely limited to the extremities. Nevertheless, the SAT of the lower abdomen is frequently claimed to be symptomatic in lipedema (18). The involvement of abdominal adipose tissue depots in the characteristic fibrotic remodeling processes remains uncertain. Here, we investigated the characteristics of lipedema stages in the unaffected abdominal region in comparison to the thigh regions of lipedema patients, which feature clear signs of pathology, as well as age- and body mass index (BMI)-matched control subjects without lipedema to enable a better understanding of definition of clinically determined disease stages. Moreover, we show that distinct molecular parameters, such as adipocyte size, thigh SAT fibrosis, and macrophage accumulation, emerge in a stage-dependent manner and could be used as diagnostic tools to better characterize patients and develop adequate treatment options.

## 2 Materials and methods

### 2.1 Human subjects

The study protocol was approved by the local ethics committee prior to patient enrollment (Nr. S 6(a)/2020, accepted on 18. February 2020) and the study was conducted according to the principles of the Declaration of Helsinki and its amendments. All subjects provided their informed consent before study participation. Lipedema was diagnosed according to the criteria described in our previous work (1), including (1) pain upon pressure and touch in the affected limbs, (2) negative Stemmer’s sign, (3) bilateral, symmetrical, disproportionate adipose tissue hypertrophy on the limbs, (4) easy bruising, and (5) sparing of the feet. For study inclusion, the diagnosis for all lipedema patients had to be confirmed by an independently practicing non-surgical physician. Conservative treatment methods must have been applied for at least 6 months prior to surgery (19). A total of 32 female patients were included in the study cohort which fulfilled the criteria, of which 9, 16, and 7 individuals could be assigned to the progredient lipedema severity stages I, II and III, respectively (**Table 2A**). The control group consisted of 14 women who did not report lipedema-associated symptoms or met any of the exclusion criteria and who underwent aesthetic liposuction of the extremities and abdomen. Individuals were age- and BMI-matched to the lipedema group. Exclusion criteria were: Previous surgery on the extremities (e.g. Varicose vein stripping, liposuction) or the abdomen (e.g. laparotomy), diabetes of any kind, severe uncontrolled endocrine, cardio-vascular, pulmonary or inflammatory/rheumatic diseases, moderate or severe psychiatric diseases of any kind, lymphedema according to the German Lymphedema Guidelines, current skin infections or other signs of acute inflammation, pregnancy, primary obesity without disproportion or secondary obesity (BMI > 35kg/m²), fat distribution disorders of other genesis (e.g., painless lipohypertrophy, benign symmetric lipomatosis) and fibromyalgia.

**Table 2:** Study details of control and lipedema patients classified to lipedema stages I-III.

	LIPEDEMA	CONTROL	P-VALUE*
(A)TOTAL COHORT			
SAMPLE SIZE			
All patients	n = 32	n = 14	
Stage I	n = 9		
Stage II	n = 16		
Stage III	n = 7		
BMI IN KG/M²			
All patients	28.89 ± 4.00	26.70 ± 4.82	0.1498
Stage I	25.13 ± 3.26		
Stage II	29.90 ± 3.03		
Stage III	31.42 ± 3.73		
AGE IN YEARS			
All patients	37.44 ± 12.38	38.71 ± 9.93	0.7131
Stage I	31.56 ± 8.86		
Stage II	37.31 ± 12.86		
Stage III	45.29 ± 12.22		
(B)HE/SR STAINING			
SAMPLE SIZE			
All patients	n = 30	n = 14	
Stage I	n = 9		
Stage II	n = 16		
Stage III	n = 5		
BMI IN KG/M²			
All patients	28.66 ± 3.99	26.70 ± 4.82	0.1983
Stage I	25.13 ± 3.26		
Stage II	29.90 ± 3.03		
Stage III	31.03 ± 4.27		
AGE IN YEARS			
All patients	37.30 ± 12.54	38.71 ± 9.93	0.6893
Stage I	31.56 ± 8.86		
Stage II	37.31 ± 12.86		
Stage III	47.60 ± 12.46		
(C)QPCR GENE EXPRESSION			
SAMPLE SIZE			
All patients	n = 32	n = 14	
Stage I	n = 9		
Stage II	n = 16		
Stage III	n = 7		
BMI IN KG/M²			
All patients	28.89 ± 4.00	26.70 ± 4.82	0.1498
Stage I	25.13 ± 3.26		
Stage II	29.90 ± 3.03		
Stage III	31.42 ± 3.73		
AGE IN YEARS			
All patients	37.44 ± 12.38	38.71 ± 9.93	0.7131
Stage I	31.56 ± 8.86		
Stage II	37.31 ± 12.86		
Stage III	45.29 ± 12.22		
(D)IMMUNOSTAINING			
SAMPLE SIZE			
All patients	n = 18	n = 12	
Stage I	n = 6	n = 4	
Stage II	n = 7	n = 4	
Stage III	n = 5	n = 4	
BMI IN KG/M²			
All patients	27.03 ± 4.14	26.56 ± 4.15	0.7660
Stage I	23.46 ± 2.38	22.52 ± 1.41	0.4579
Stage II	27.23 ± 2.36	25.94 ± 1.48	0.2952
Stage III	31.03 ± 4.27	31.23 ± 2.74	0.9350
AGE IN YEARS			
All patients	38.22 ± 12.97	40.58 ± 9.19	0.5643
Stage I	30.67 ± 8.64	35.00 ± 4.76	0.3388
Stage II	38.00 ± 13.43	42.50 ± 12.01	0.5850
Stage III	47.60 ± 12.46	44.25 ± 8.85	0.6522
(E)BLOOD PLASMA ANALYSES			
SAMPLE SIZE			
All patients	n = 31	n = 14	
Stage I	n = 8		
Stage II	n = 16		
Stage III	n = 7		
BMI IN KG/M²			
All patients	29.06 ± 3.94	26.70 ± 4.82	0.1226
Stage I	25.31 ± 3.44		
Stage II	29.90 ± 3.03		
Stage III	31.42 ± 3.73		
AGE IN YEARS			
All patients	37.58 ± 12.55	38.71 ± 9.93	0.7470
Stage I	31.38 ± 9.46		
Stage II	37.31 ± 12.86		
Stage III	45.29 ± 12.22		

(A) Details about the total cohort of lipedema and control patients with characteristic values for BMI [kg/m^2^] and age [years]. (B-E) Baseline characteristics BMI [kg/m^2^] and age [years] for controls and matched total lipedema group as well as parameters for lipedema stage groups for each experimental setup: (B) analysis of adipocyte size with HE and Sirius red for determination of fibrosis area, (C) gene expression analysis, (D) immunohistochemistry for macrophage analysis and (E) plasma lipid profiles. Data are presented as mean ± SD. *using unpaired, two-tailed t-test with Welch correction.

### 2.2 Tissue collection

Adipose tissue biopsies were obtained from the thigh and the lower abdomen regions of female lipedema patients and age- and BMI-matched control subjects during elective liposuction. Tissue samples of the thigh were harvested in the ventral portion of the dominant affected (or right) thigh midway between the anterior superior iliac spine and the cranial margin of the patella. Samples from the caudal abdomen were taken originating from the umbilical approach. The biopsies were harvested before infiltration of the tumescent solution by surgical dissection through the regular surgical incisions for liposuction. One-half of the freshly obtained biopsy samples was stored in phosphate-buffered saline (PBS) with 3.5% BSA (Sigma-Aldrich, Taufkirchen, Germany) at 4°C immediately after collection and during transport. Subsequently, the specimens were fixed in 4% formaldehyde/PBS before embedding for histology. The remaining biopsy material was immediately transferred into dry 1.5 ml collection tubes and stored without addition of cryopreservant on dry ice. For long-term storage, frozen biopsy specimens and lipoaspirates were transferred to -80°C.

### 2.3 Histology and immunostaining

Adipose tissue samples of 14 controls and 9 stage I, 16 stage II and 5 stage III classified lipedema patients (**Table 2B**) were fixed in 4% paraformaldehyde (Carl Roth GmbH, Karlsruhe, Germany) for 24 hours at 4°C, dehydrated embedded in paraffin, and cut into 4-µm sections. To determine adipocyte diameter, area and number, sections were stained with hematoxylin and eosin (H&E) staining. Slices were photographed at 100x magnification and analyzed with Image J software (NIH, Bethesda, MA, USA) as described elsewhere (20). A total of three sections per patients and 2 non-overlapping fields per section were assessed. To determine fibrosis area, Sirius red staining was performed in adipose tissue deparaffinized slides incubated with a 0.1% Sirius red solution dissolved in aqueous saturated picric acid for 1 hour, washed in acidified water (0.5% acetic acid) and mounted for image analysis. All images were captured at 200x magnification and fibrosis area [μm^2^] was quantified using Image J software (NIH, Bethesda, MA, USA) with the freely available plugins “MRI fibrosis tool” and “Color Deconvolution” (21). A total of three sections per patient and 3 different non-overlapping fields per section were quantified (**Table 2D**).

For immunohistochemistry, a meticulous selection process was implemented to minimize the potential influence of age and BMI as factors affecting macrophage numbers. Initially, the 14 control samples were divided into three groups, ensuring equitable distribution while excluding the samples with the lowest and highest BMI values. Subsequently, from each stage, six lipedema patients with the lowest BMI were selected to match the control subgroups in terms of age and BMI. Regrettably, due to substantial damage during the paraffin embedding process, only five sections from stage III lipedema patients were accessible. As a solution, a matched stage II patient was included in the analysis to maintain consistency and comparability. The paraffin-embedded sections were deparaffinized and incubated with antibodies to pan-macrophage (anti-CD68, Abcam,Cambrige, United Kingdom), M1-like macrophages (anti-CD86, Abcam, Cambrige, United Kingdom), and M2-like macrophages (anti-MRC1/CD206, Abcam, Cambrige, United Kingdom) at 4°C overnight. Secondary antibodies were either Anti-rabbit (AlexaFluor-594-labelled, Thermo Fisher Scientific, Dreieich, Germany) or Anti-mouse (AlexFluor-488-labelled, Thermo Fisher Scientific, Dreieich, Germany). 4’,6-Diamidino-2-Phenylindole, Dihydrochloride (DAPI, Biolegend, London, United Kingdom) was used for nuclei staining. Images were captured at 200x, and labelled cells were counted manually using BZ-II Viewer software (Keyence, Ōsaka, Japan).

### 2.4 RNA extraction and gene expression analysis

RNA from 30mg of minced and frozen adipose tissue samples (**Table 2C**) was homogenized using a bead-based micro-blender with in QIAzol lysis reagent (Qiagen, Hilden, Germany for 5 min at 4°C followed by centrifugation for 10min at 4°C and 12,000 rpm to separate lipid layer from aqueous extract. Phase separation was initiated by adding 100 µl chloroform. After 15 min of centrifugation at 12,000 rpm and 4°C, RNA was precipitated from the upper clear phase with 250 µl of isopropanol. RNA was purified with 500 µl of 75% ethanol, dried and solubilized in 30 µl sterile-filtered water treated with di-ethyl-pyrocarbonate (DEPC). Purified RNA was reversely transcribed into cDNA using a high-capacity cDNA reverse transcription kit (Thermo Fisher Scientific, Dreieich, Germany). Quantitative real-time PCR was performed using Maxima SYBR Green/ROX qPCR Master Mix (Thermo Fisher Scientific, Dreieich Germany) on a CFX384 Touch instrument (Bio-Rad, Munich, Germany). Primers were designed as intron spanning sequences to specifically amplify cDNA (primer sequences: **Supplementary Table S1**). The mRNA expression levels were determined using the 2^(–ΔΔCT) method and normalized to mRNA levels of human beta actin (hACTB) as housekeeping gene.

### 2.5 Plasma isolation and analysis

For the assessment of the metabolic risk profile, blood plasma samples were collected preoperatively after 6-8 h of starvation in an S-monovette (Sarstedt, Nuernbrecht, Germany). Blood samples were stored on ice until further processing, subsequently centrifuged for 10 min at 6,000 rpm at 4°C, after which plasma was collected and stored at -80°C until analysis. Plasma profiles of 14 controls were analyzed and compared to 8 stage I, 16 stage II and 7 stage III classified lipedema patients (**Table 2E**). Plasma insulin was determined using a human insulin ll ELISA kit (BioVendor R&D, Czech Republic) according to the supplied protocol. Blood parameters were measured on a Cobas Mira Analyzer (Roche Diagnostics, Mannheim, Germany) by using commercially available assay kits for free fatty acids (FFA), (Wako chemicals, Neuss, Germany), glycerol (Randox, Crumlin, UK), triglycerides, cholesterol, high- density lipoprotein (HDL), and glucose (Axonlab, Stuttgart, Germany).

### 2.6 Statistical analysis

Analyses were performed using GraphPad Prism, version 8.0.0 for Windows (GraphPad Software, San Diego, CA, USA). All data were presented as mean ± standard error of the mean (SEM), or median (interquartile range, 25-75%). Sample sizes as well as the statistical analyses are indicated in the respective figure legends. Welch corrected t-test and multiple t-tests were applied for comparisons between two groups. Statistically significant differences are assumed for p-values < 0.05.

## 3 Results

### 3.1 Stage dependent adipocyte hypertrophy in lipedema occurs predominantly in thigh regions

After verification of previously published diagnostic criteria (1) and evaluation of exclusion criteria, a total of 32 lipedema patients were recruited in our study and divided into severity stages (stage I: n=9; stage II: n=16; stage III: n=7). Moreover, 14 controls subjects were enrolled that were matched to the patient cohort for BMI (**Table 2A**; control: 26.7 ± 4.8 kg/m² versus lipedema: 28.9 ± 4.0 kg/m², p=0.1498) and age (**Table 2A**; control: 38.7 ± 9.9 versus Lipedema: 37.4 ± 12.4, p=0.7131).

**Figure 1:**
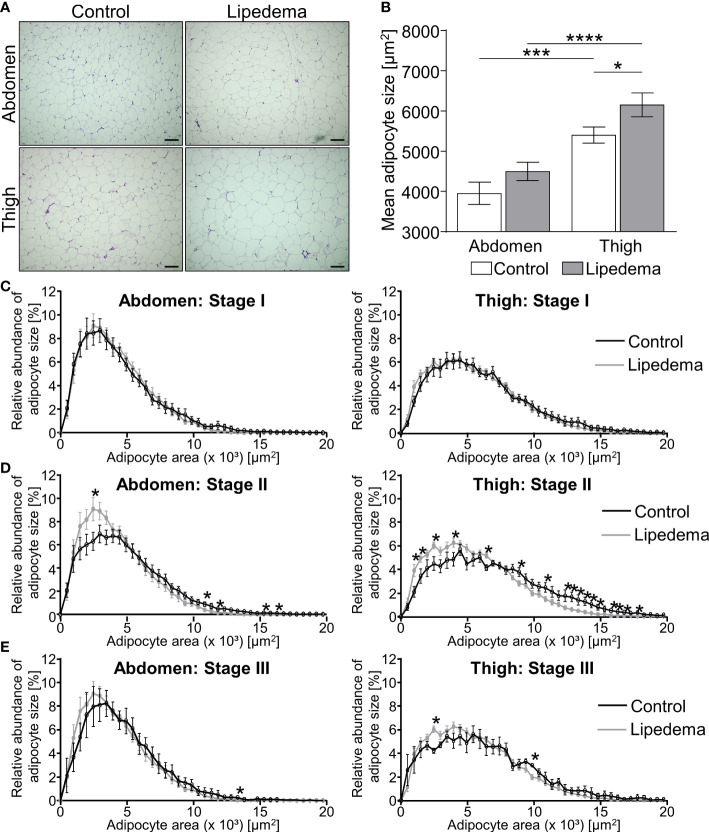
Adipocyte hypertrophy occurs in later stages of lipedema progression. **(A)** Representative images of hematoxylin and eosin (H&E) staining of adipose tissue sections from the abdominal and thigh regions of lipedema patients and BMI and age matched controls (100x magnification; scale bar 100 µm). **(B)** Quantification of average adipocyte size, expressed as area in µm^2^, in lipedema patients (n = 30; grey bars) and control subjects (n=14; white bars) in the abdominal and thigh regions. **(C–E)** Adipocyte size distribution in abdominal region (panels on left and thigh (panels on right) in the indicated lipedema stage I [black lines; n=9; **(C)**], stage II [n= 16; **(D)**] and stage III [n=9; **(E)**] as percentage of total counted number of cells, compared to the same group of control subjects in each panel (n=14; gray line). Representative images of stage dependent H&E stained adipose tissue sections are depicted in **Supplementary Figure S1**. Data are presented as mean ± SEM. Statistical significances are indicated as *p<0.05; ***p<0.0005; ****p<0.0001 using unpaired, two-tailed t-test with Welch correction for B and nonparametric multiple t-test for **(C–E)**.

Adipose tissue morphology was assessed in thigh and abdominal biopsies for all patients included in this study (stage I: n=9; stage II: n= 16; stage III: n=5) and compared to morphologies of the 14 non-lipedema control subjects (**Table 2B**). In general, individual adipocyte size was larger in thigh biopsies compared to abdominal regions in both groups. In lipedema patients a significantly elevated average adipocyte size was observed in the thigh region while no differences were detected in abdominal biopsies (**Figures 1A, B**).

The stage dependent analysis revealed no significant differences between lipedema and controls in both biopsies comparing stage I patients to the control group (**Figure 1C**, **Supplementary Figure S1**). Conversely, a higher abundance of hypertrophic adipocytes in thigh and abdominal biopsies of lipedema patients of stage II was observed, showing elevated counts of smaller adipocytes and reduced numbers of large-area adipocytes in biopsies of the control group (**Figure 1D**, **Supplementary Figure S1**). In stage III lipedema patients, a similar pattern emerged, altogether shifting adipocyte distribution towards larger cells in lipedema thigh regions, albeit not reaching statistical significance in the abdominal samples (**Figure 1E**, **Supplementary Figure S1**).

In summary, the phenotypical changes of the pathology were most pronounced in stage II and III patients, in comparison to the control group and stage I patients. A potential explanation for the seemingly milder phenotype in stage III is that this subgroup of patients also presented with the highest BMI (**Table 2B**). This distinction could act as confounding factor resulting in increased adipocyte sizes in all depots of stage III patients independently of lipedema itself.

### 3.2 Progressive fibrosis corresponds to lipedema stage severity

**Figure 2:**
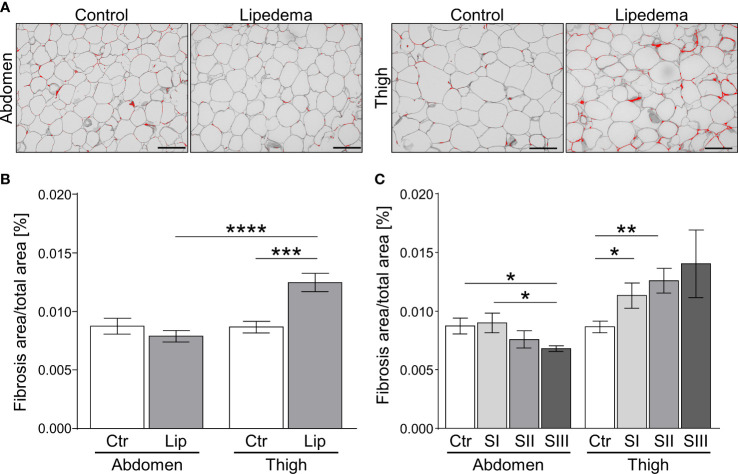
Fibrosis occurs as an early sign of lipedema and presents only in SAT of affected extremities. **(A)** Representative images of Sirius red-stained interstitial fibrosis in abdomen and thigh SAT sections (200x magnification; scale bar 100 µm) in lipedema patients and control subjects matched for age and BMI. **(B)** Quantification of fibrosis areas as percentage of total area comparing sections derived from controls (n=14; white bars) and lipedema patients (n=30; grey bars). **(C)** Stage dependent analysis of fibrosis area as percentage of total area in control patients (n=14; white bars) compared to stage I (SI; n=9; light grey), stage II (SII; n=16; grey), stage III (SIII; n=5; dark grey) lipedema patients in sections of abdominal and thigh adipose tissue. Representative images of stage dependent H&E stained adipose tissue sections are depicted in **Supplementary Figure S2**. Data are presented as mean ± SEM. *p<0.05; **p<0.005; ***p<0.0005; ****p<0.0001 using unpaired, two-tailed t-test with Welch correction.

The appearance of hypertrophic adipocytes is frequently accompanied by remodeling of the extracellular matrix (ECM). We therefore conducted a histological analysis of fibrosis levels in controls and lipedema patients. No difference of pericellular fibrosis was detected in the non-symptomatic lower abdominal region when comparing the control group to all lipedema patients while a significant increase of fibrotic areas was detected in the lipedema-affected thigh compared to healthy controls (**Figures 2A, B**). Evaluation of stage-dependent fibrosis levels in the abdominal region overall confirmed no evidence for elevated fibrosis in any stage, but unexpectedly revealed significantly lower fibrosis levels in stage III (**Figure 2C**). In contrast, a significant stage-dependent increase of fibrosis was found in stage I and II patients and a similar trend was observed in stage III when comparing histologies of thigh biopsies to the control group (**Figure 2C**, **Supplementary Figure S2A**).

Taken together, our findings show that pathological ECM-remodeling of thigh, but not abdominal adipose tissue depots manifests early on in the disease, i.e. at stage I, thus preceding adipocyte hypertrophy and altogether suggesting that this aspect of lipedema warrants further investigation as a marker with diagnostic value.

### 3.3 Upregulation of inflammatory marker genes in lipedema-affected regions

**Figure 3:**
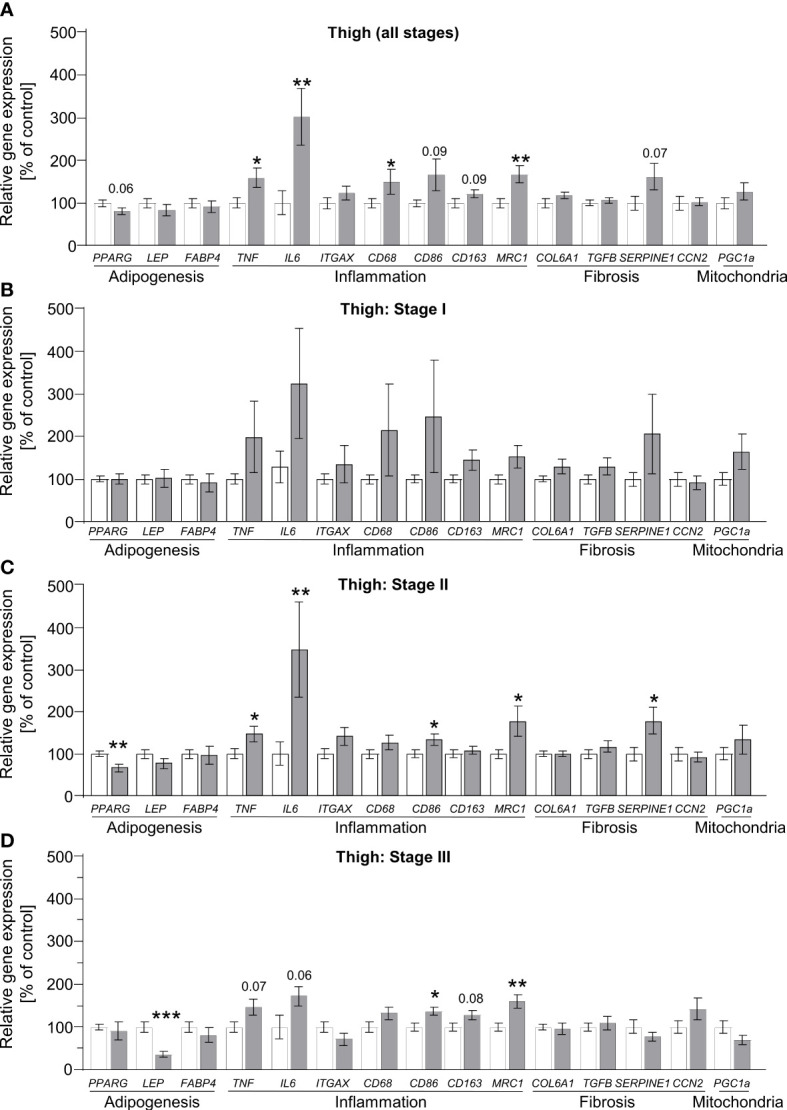
Stage-dependent gene expression patterns show increased inflammation-linked processes in thigh SAT of lipedema patients. **(A)** Gene expression analysis of marker genes related to adipogenesis, inflammation, fibrosis and mitochondrial function in thigh SAT of lipedema patients (n=32, grey bars) depicted as relative gene expression of all non-lipedema control subjects (n=14, white bars). **(B)** Analysis similar to panel A comparing all controls (n=14; white bars) to stage I lipedema thigh SAT (n=9; grey bars). **(C)** Analysis similar to panel A comparing all controls (n=14; white bars) to stage II lipedema thigh SAT (n=16; grey bars). **(D)** Analysis similar to panel A comparing all controls (n=14; white bars) to stage III lipedema thigh SAT (n=7; grey bars). All data are represented as mean ± SEM. *p<0.05; **p<0.005; ***p<0.0005 using unpaired, two-tailed t-test with Welch correction.

To define alterations of adipose tissue morphology in lipedema on the transcriptional level, we measured the gene expression profiles of markers reflecting the processes of adipogenesis, inflammation, fibrosis and angiogenesis. Additionally, the marker peroxisome proliferator-activated receptor gamma-coactivator 1 alpha (*PPARGC1A*, also known as *PGC1A*) was assessed as a general marker of mitochondrial function. In abdominal and thigh biopsies comparing control subjects to all lipedema patients, several marker genes of inflammation and angiogenesis were broadly and significantly upregulated, suggesting that this process is a target of lipedema pathology (**Figure 3A**, **Supplementary Figures S3**, **S4**). We subsequently re-analyzed the data by comparing the control group to the three stage groups individually. Here, no statistically different gene expression was recorded at stage I in the lipedema-affected thigh region except for hypoxia-inducible factor 1-alpha (*HIF1A*, **Supplementary Figure S4**), while expression of inflammatory marker gene interleukin-6 (*IL6*) and fibrosis marker collagen 6a1 (*COL6A1*) were elevated in abdominal biopsies of stage I patients (**Figure 3B**, **Supplementary Figure S3B**). In abdominal biopsies, these changes, in particular pertaining to *IL6*, remained relatively stable throughout the more severe stage II, but only limited, if any, further differences became apparent, which was consistent with the relatively mild changes in the all group-comparison of abdominal biopsies (**Supplementary Figure S3**). Conversely, thigh biopsies started featuring several significantly differentially expressed genes in stages II and III, such as reduced expression of the adipokine leptin (*LEP*), and increased expression of angiogenesis marker gene vascular endothelial growth factor C (*VEGFC*) **Supplementary Figures S4C, D**) and several inflammatory marker genes, including *IL6*, tumor necrosis factor-alpha (*TNF*), and macrophage markers *CD86* and mannose receptor c-type 1 (*MRC1*; also known as *CD206*) (**Figures 3C, D**). In none of the conditions, *PPARGC1A* (**Figure 3**) expression was altered, suggesting that mitochondrial biogenesis is normal during lipedema disease progression. In summary, these data suggest that non-affected adipose tissue regions, such as the abdominal depot, may display altered gene expression patterns as an early indicator of lipedema, whereas expression of inflammatory markers genes is stage-dependent in affected adipose depts of the extremities and correlates with disease severity. Decreased expression of leptin also suggests an altered endocrine profile of the affected adipose thigh regions.

### 3.4 Stage-dependent alterations of adipose-tissue macrophage populations in lipedema

**Figure 4:**
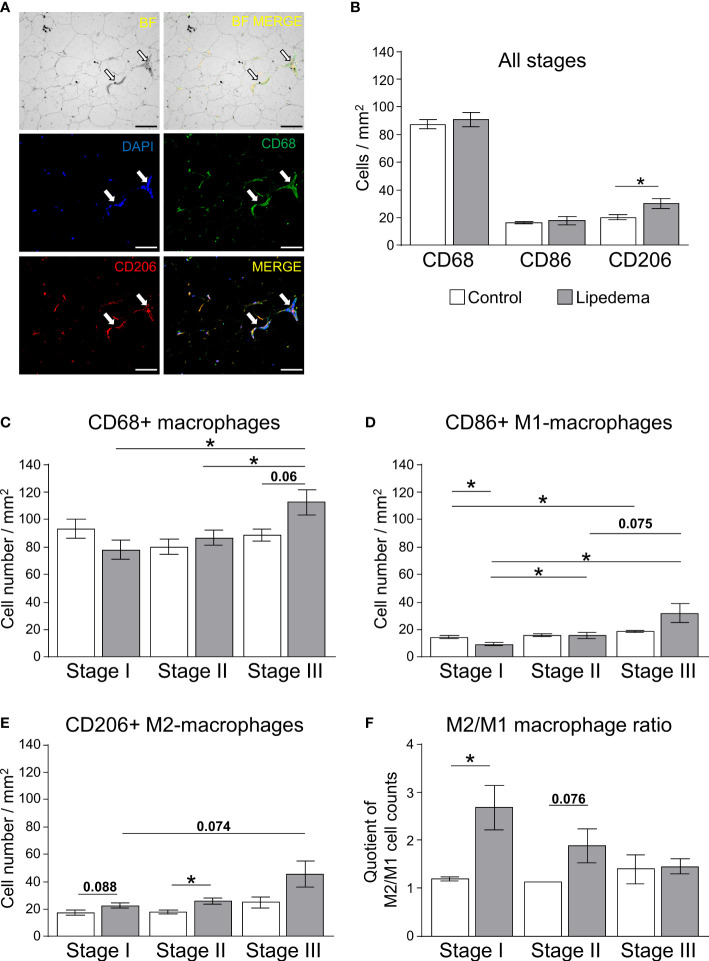
Impairment of macrophage polarization during pathogenesis of lipedema. **(A)** Representative bright field analysis (BF) and bright field merged with immunofluorescent signals (BF MERGE) of macrophage infiltration in thigh adipose tissue of lipedema patients. Representative images of immunofluorescence staining of macrophages in lipedema patient to detect DAPI nuclei (blue), CD68+ pan-macrophage (green) and CD206+ anti-inflammatory macrophage (red) markers Merged: yellow indicates co-staining of anti-CD68 and anti-CD206 antibodies). White arrows indicate crown-like-structures (200x magnification; scale bar 100 µm) **(B)** Quantification of macrophage cell counts per area [mm²] comparing thigh SAT of lipedema patients (n=18, grey bars) and controls (n=12; white bars) matched for age (p=0.5643) and BMI (p=0.7660). Quantification summarizes counts per area for pan-macrophages expressing CD68, M1-like macrophages expressing CD86, and M2-like macrophages expressing CD206. **(C–E)** Quantification of stage-specific macrophage cell counts per area (mm²) in lipedema patients according to disease stage (stage I: n=6, stage II: n=7; stage III: n=5; grey bars) and compared to stage-specific control subgroups matched for BMI and age (n=4 for each stage-specific control group; white bars) depicting CD68+ pan-macrophages **(C)**, CD86+ pro-inflammatory M1-like macrophages **(D)**, and CD206+ anti-inflammatory M2-like macrophages **(E)**. **(F)** Calculated M2/M1 ratios depending on disease stage (grey bars) and respective control subgroup (white bars). All data are represented as mean ± SEM. *p<0.05 using unpaired, two-tailed t-test with Welch correction.

To corroborate the increased gene expression markers associated with inflammation and adipose tissue macrophages in thigh adipose tissue of lipedema patients, immunohistochemistry of CD68+ pan-macrophages, CD86+ M1-like (pro-inflammatory) macrophages (**Supplementary Figure S5A**), and CD206+ M2-like (anti-inflammatory) macrophages were performed (**Figure 4A**, **Supplementary Figure S5B**). Crown-like structures (CLS) as hallmarks of proinflammatory processes in adipose tissue were present in all stages of lipedema (**Figure 4A**), but no significant differences in the total numbers of CD68+ and pro-inflammatory M1 macrophages were detected when comparing all patients to the control group (**Figure 4B**). In contrast to this, the number anti-inflammatory M2-like macrophages, expressing the surface marker CD206 was significantly increased in lipedema patients in the stage-independent analysis (**Figure 4B**) As before, this analysis was re-assessed by comparing the control subjects to the subgroups representing stages I through III. For this analysis, age- and BMI-matched individuals were selected from the control group for comparisons to rule out these two parameters as potential determinants of altered macrophage numbers. Although no significant differences regarding total macrophages were evident in the all group-comparison, we detected a progressive increase of CD68+ macrophages from stage I o stage III whereas no such increase was found in the matched control subsets (**Figure 4C**). The staining for specific macrophage subtypes revealed a stage-dependent increase of M1-like macrophages in lipedema patients from stage I to stages II and III. This effect was less pronounced in the control groups, where the stage I control group only differed significantly from the stage III control group, presumably due to the increase in BMI between these subgroups (**Figure 4D**, **Table 2D**). As a result of this trend in patients, M1-macropage levels were significantly reduced at stage I compared to the control group and a marked trend was found for increased M1-levels at stage III compared to the control group (**Figure 4D**). Numbers of CD206+ anti-inflammatory, M2-like macrophages were found to be increased in thigh adipose biopsies of stage II lipedema patients compared to the matched control groups, again confirming the observations form the gene expression and all group-comparison analyses (**Figures 4B, E**). As expected, the same stage-correlating increase of anti-inflammatory macrophages from stage I to III lipedema was found, while no alterations in the control subgroups were found (**Figure 4E**). In summary, a stage-dependent increase of macrophage levels in lipedema was observed while cell numbers remained relatively stable in age- and BMI-matched controls assigned to the respective disease stages. Somewhat unexpectedly, paralleling trends towards increased M1- and M2-macrophage accrual was detected in this analysis. Calculating rations of the two distinct macrophage subsets (M2:M1) is a tool frequently used to assess whether pro- versus anti-inflammatory signals are more prevalent in the adipose tissues. Accordingly, the M2:M1 macrophage ratio was significantly increased in stage I patients compared to the ratio for matched controls, and showed a similar trend (p=0.076) in stage II patients (**Figure 4F**). Conversely, no difference in the ratios of stage III patients and matched controls was evident, altogether suggesting a stage-dependent decline of the M2-driven anti-inflammatory milieu in late-stage disease (**Figure 4F**).

### 3.5 Distinct lipid profile of lipedema patients

**Figure 5:**
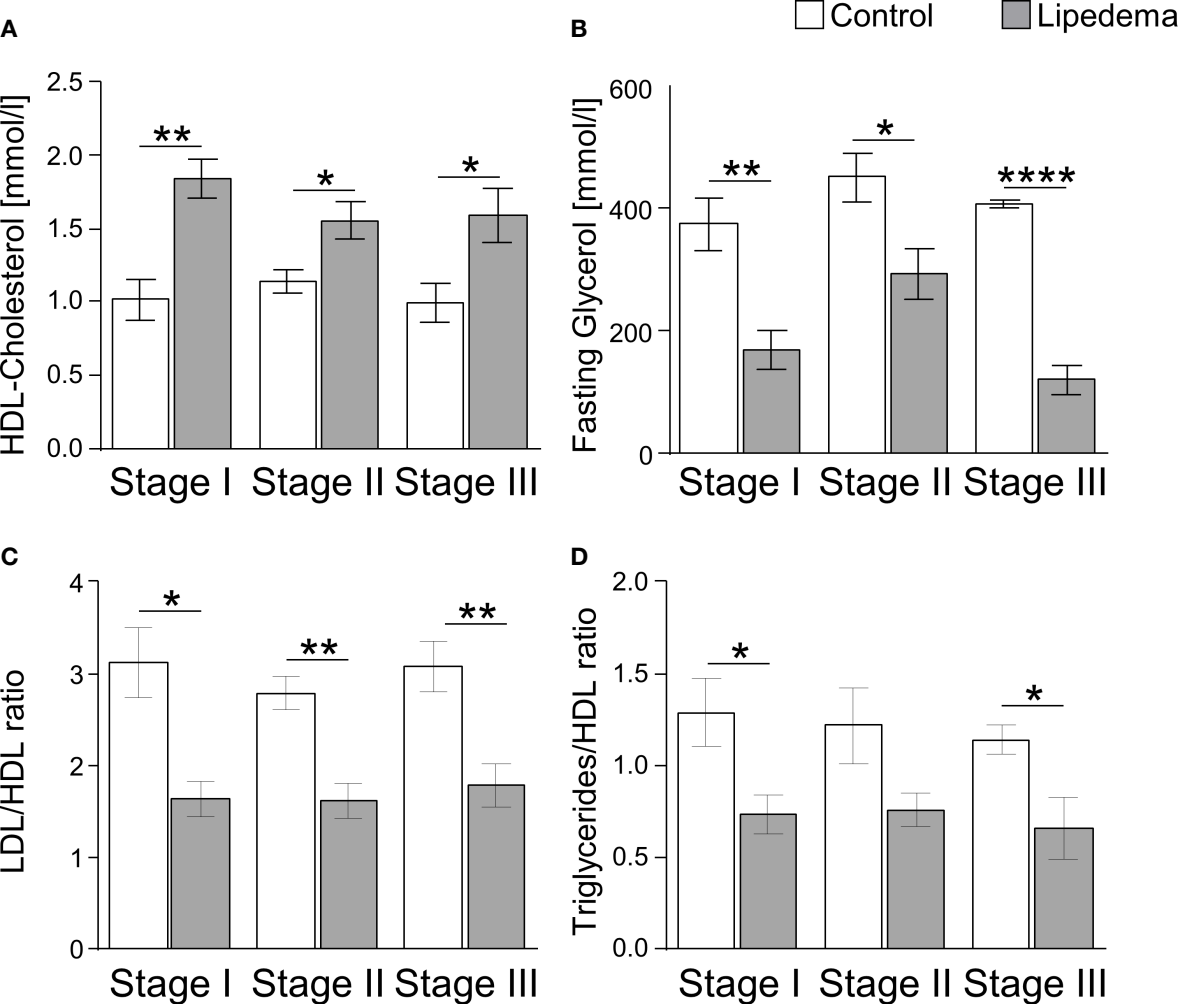
Lipedema-associated changes of plasma lipid profiles. **(A)** Analysis of plasma levels of HDL-Cholesterol comparing controls (white bars) to lipedema patients (grey bars) of stage I (n=8), stage II (n=16) and stage III (n=7). **(B)** Analysis of plasma levels of fasting glycerol comparing controls (white bars) to lipedema patients (grey bars) of stage I (n=8), stage II (n=16) and stage III (n=7). **(C)** Calculation of the quotient between low-density lipoprotein (LDL) and high-density lipoprotein (HDL) for estimation of metabolic risk in controls (white bars) and lipedema patients (grey bars). LDL concentration was estimated by the Friedewald equation: [total cholesterol] – [high-density lipoprotein cholesterol] –[triglycerides/5]. **(D)** Calculation of the quotient between triglycerides and HDL comparing controls (white bars) to lipedema patients of stage I to stage III (grey bars). *p<0.05; **p<0.005; ****p<0.0001.

As lipedema-associated changes in our cohort, such as adipocyte hypertrophy, interstitial fibrosis, and inflammation, are commonly associated with an altered metabolic profile in patients with primary obesity, we analyzed plasma samples to assess glucose and lipid metabolism in patients and controls (**Table 3**). While no differences were found in parameters reflecting glucose homeostasis, including the homeostatic model assessment of insulin resistance (HOMA-IR), the plasma lipid profile displayed several differences. Total cholesterol and triglyceride levels were found to be normal in lipedema patients, but elevated high-density lipoprotein (HDL) cholesterol and reduced plasma glycerol were detected. In addition, the low-density lipoprotein (LDL)/HDL cholesterol and the triglyceride/HDL ratios, representing potential predictors for the risk of metabolic diseases, were lower in lipedema patients. The group breakdown into disease stages and the corresponding control subgroups led to loss of statistical significance, but the data show that these lipedema-associated changes in plasma lipids were robust and evident at all stages of disease severity, pertaining to HDL, glycerol and the LDL/HDL and triglycerides/HDL ratios, while no additional stage-specific differences occurred in any of the other parameters (**Figure 5**, and not shown).

**Table 3:** Lipedema correlates with improved plasma lipid profiles.

	Lipedema	Controls	p-value
Total cholesterol (mmol/l)	4.42 ± 1.07	4.29 ± 0.98	0.7023
Triglycerides (mmol/l)	1.07 ± 0.40	1.244 ± 0.42	0.2163
High-density lipoprotein (HDL) (mmol/l)	1.65 ± 0.46	1.04 ± 0.27	**<0.0001**
Low-density lipoprotein (LDL) (mmol/l)*	2.55 ± 0.84	3.00 ± 0.77	0.0901
Non-HDL cholesterol (mmol/l)	2.77 ± 0.87	3.25 ± 0.77	0.0719
LDL : HDL ratio	1.65 ± 0.65	2.96 ± 0.62	**<0.0001**
Triglycerides : HDL ratio	0.72 ± 0.36	1.21 ± 0.33	**0.0001**
Free fatty acids (mmol/l)	0.74 ± 0.15	0.70 ± 0.29	0.5855
Plasma glycerol (mmol/l)	219.00 ± 143.90	403.10 ± 75.83	**<0.0001**
Fasting plasma glucose (mmol/l)	5.13 ± 0.74	4.71 ± 0.66	0.0710
Fasting plasma insulin (mmol/l)	3.86 ± 3.08	4.52 ± 1.71	0.3993
HOMA-IR**	0.83 ± 0.65	0.95 ± 0.37	0.4848

Markers of lipid and glucose metabolism in blood plasma comparing lipedema patients (n=31) and controls (n=14) matched for age (p=0.7470) and BMI (p=0.1226). Data are presented as mean ± SD, and p<0.05 is considered statistically different (*estimated by the Friedewald equation: [total cholesterol] – [high-density lipoprotein cholesterol] –[triglycerides/5]); **HOMA-IR= Homeostatic model assessment of insulin resistance).

Bold values highlight statistically significant p-values.

## 4 Discussion

The progression of lipedema has been linked to three pathophysiological processes, (1) morphological changes to adipocytes in the affected adipose depots, (2) accumulation of fibrotic areas within adipose depots, and (3) inflammatory processes, which are characterized by altered immune cell distribution and cytokine release patterns. A major challenge to adequate medical care for patients with lipedema is a robust and early diagnosis of the disease. In the present study, we have therefore combined diagnostic staging of disease severity, which requires specialist expertise in the attending physician, with a morphological assessment of adipose tissue and molecular analysis of fibrosis and inflammation. We show that all pathological changes occur stage-dependently and that adipocyte hypertrophy only occurs as a marker of later and more severe disease stages. It is preceded by characteristic changes in tissue fibrosis, inflammatory marker gene expression, and macrophage accumulation. Moreover, our data also show that patient-reported discomfort, which is mainly localized to SAT of the extremities, can be supported by objective measurement readouts. However, it does not serve as a certain predictor of molecular changes, which also occur in SAT of the abdominal region without a clear association to lipedema pathology, albeit with a milder phenotype. In summary, our analysis establishes that biomarkers of disease could help improve the initial diagnosis.

Adipocyte hypertrophy and heterogeneity in cell size in lipedema thighs was previously reported by several groups (7, 9–11, 15). Interestingly, the results differed in some details: While an average cell area of 7,000-8,000 µm² was reported in a cohort with predominantly lipedema stage I and II patients (7), cell areas of more than 12,000 µm² were found in populations with predominantly lipedema stage II and III patients (10, 11). With approximately 6,000 µm², and factoring in technical differences, the median cell areas observed in our study were in a comparable range to the results of Al-Ghadban et al. and confirm the reported heterogeneity of cell sizes while providing evidence of a stage-dependent shift towards larger cell sizes (7). The limited significance of the size shift we observed in stage III patients might be due to a concomitant increase in BMI at this stage and could therefore reflect the possible influence of secondary obesity rather than a stage III-specific effect. Consistent with previous studies, we show that the adipocyte cell size was larger in thigh SAT biopsies compared to abdominal SAT biopsies in both lipedema patients and controls (22). In our study, the lipedema affected thigh regions generally showed more severe changes compared to the abdominal SAT, although mild adipocyte hypertrophy was also observed.

Similar to fat mass expansion in obesity, our study demonstrated increased amounts of collagen as a sign of pericellular fibrosis surrounding adipocytes in lipedema (23). In contrast to primary obesity, the pathological accumulation of collagenous fibrosis areas was only found in the clinically affected extremities but not in the abdomen of lipedema patients. Thus, the clinical assessment of tissue texture as part of the staging classification is reflected in the objective histologic assessment. Our patient cohort reported the main medical discomfort as being localized to the extremities. However, reports exist that the abdomen may exhibit lipedema-like texture, i.e. nodule-structures were found in abdominal SAT in 24% of patients, and other symptoms such as pain in these areas was reported by 14% of patients (18, 24).

Given that fibrosis was significantly elevated already in stage I patients, i.e. prior to onset of adipocyte hypertrophy, we believe such alterations could serve as markers for lipedema but might also be linked to the subsequently occurring changes in adipocyte size. While a general shift in adipocyte distribution toward larger cells was observed in the lipedema thigh regions in stage II and III, the mean adipocyte size did not vary between the stages. Some reports suggest that excessive extracellular matrix remodeling in the form of fibrosis inversely correlates with decreased adipocyte size, meaning that increased adipose tissue fibrosis restrains adipocyte hypertrophy (23, 25–27). This may be an explanation for the increase in interstitial fibrosis in stage I lipedema, even though adipocyte size is largely normal.

It is generally accepted that modulation of inflammation may play a key role in the pathogenesis of lipedema (7–9, 11, 13, 28). Still, it remains unclear to what extent this is a causal relationship that drives adipose tissue dysfunction and local SAT remodeling or a consequence of hypertrophic SAT expansion and consequent adipocyte necrosis). Our analysis of different disease stages suggests that the former might be the case, as changes in adipose tissue gene expression patterns occur prior to clear evidence of adipocyte hypertrophy. Chronic, low-grade inflammation in SAT of obese humans contributes to insulin resistance and metabolic dysfunction and affects the recruitment of tissue macrophages (29). In addition, adipocyte hypertrophy in obese patients was found to correlate significantly with SAT inflammation, particularly in terms of macrophage content (30, 31) and levels of inflammation mediators, such as IL6 and TNFα (32). Lipedema also is characterized by elevated macrophages around adipocytes and the formation of crown-like structures (7, 9, 13, 15). Traditionally, macrophages have been categorized into distinct activation states with distinct profiles of cytokine expression and release. Although such categories likely reflect only the extreme ends of the phenotypic spectrum of macrophage properties, the consensus frequently settles on the two endpoints of classically (M1) and alternatively activated (M2) macrophages (33). M1-like macrophages are generally characterized by the secretion of pro-inflammatory cytokines, such as IL1β or TNFα (34–36). In contrast, M2-like macrophages were shown to rather confer anti-inflammatory responses with a corresponding cytokine profile, which in adipose tissue contributes to improved glucose uptake and insulin sensitivity (36, 37). In obesity, adipocyte hypertrophy has been associated with increases of M1 macrophages (38) and increased gene expression of proinflammatory factors, such as TNFα (32, 38–41), or IL6 (32, 39–43), and a downregulation of IL10 (40) that is linked to M2-like polarization. In lipedema-affected thigh SAT, anti-inflammatory M2-like macrophages appear to be the predominant type in stage I while in more severe stages this effect is no longer evident, suggesting a process that favors pro-inflammatory macrophages. These results are in line with previous data demonstrating overexpression of *CD163*, a marker of M2-like macrophages, in SAT biopsies of lipedema patients (13). A potential explanation for the enhanced pro-inflammatory profile at the expense of M2-like macrophages could be that adipose tissue damage accumulates in later stages, potentially further exacerbated by the elevated BMI of late-stage patients in our cohort. Of note, the clinically not lipedema-affected abdominal region displayed similar gene expression patterns of inflammation, i.e. an upregulation of *TNF* and *IL6*. Unlike in the regions affected by lipedema, these increases were milder and appeared to not be affected by disease stage. In primary obesity, increased expression of genes related to inflammation were also detected in both the abdominal and gluteofemoral SAT, but in contrast to lipedema, these depot-specific associations were significantly weaker in the gluteofemoral SAT (44). It remains to be determined whether such changes could serve as early biomarkers of disease or might provide a gene expression signature indicating elevated risk to develop lipedema.

Lastly, an altered lipid metabolism is closely associated to adipose tissue inflammatory response in states of obesity (45, 46). Specifically, cholesterol and triglyceride-rich lipoproteins might exacerbate adipocyte hypertrophy, plasma concentrations of the proinflammatory cytokine TNFα and macrophage infiltration of adipose tissue in obese patients (47, 48). In obesity, FFA release is increased in the context of the hypertrophic expansion of adipocytes promoting insulin resistance (49). Furthermore, in visceral adipose tissue, non-HDL cholesterol concentrations are positively correlated to the proportion of pro-inflammatory macrophages (50). Recent studies have reported increased plasma concentrations of total cholesterol, LDL cholesterol and triglycerides in lipedema patients (11, 51). We therefore expected corresponding differences in the lipid profile in our study cohort. However, key predictors of metabolic risk, such as total cholesterol and FFA levels, were not altered in lipedema patients. The most pronounced differences in lipedema patients were found in plasma glycerol, HDL cholesterol and LDL : HDL ratio which suggested an overall healthier metabolic profile in lipedema patients compared to the controls and also the age- and BMI-matched control subgroups.

Obesity-related adipocyte hypertrophy was demonstrated to trigger low-grade inflammation and excessive collagen deposition that would eventually lead to abnormal glucose homeostasis and systemic insulin resistance (52). In lipedema patients, in contrast, a favorable metabolic risk profile has been reported despite some similar changes in the SAT as typical pathologies linked to the metabolic syndrome, including diabetes and dyslipidemia, are underrepresented, suggesting that our results of improved lipid metabolism parameters may be fully congruent with these observations (53, 54). The favorable lipid profile could be due to the disproportionately gynoid distribution of SAT towards the lower extremities in lipedema. Thigh fat is considered more metabolically protective compared to abdominal fat and a higher proportion of subcutaneous thigh fat is associated with a healthier inflammatory profile (44, 55). In line with these observations, our analysis shows increased anti-inflammatory macrophages within the lipedema-affected regions of the extremities and a beneficial M2:M1 macrophage ratio in stage I patients. For lipedema patients, a previous study demonstrated lower glycated hemoglobin A1c (HbA1c) and higher adiponectin levels, despite higher fasting insulin concentrations and higher inflammation and oxidative stress (51). Conversely, no differences in insulin resistance or glucose metabolism were observed in our data. Interestingly, structural changes, such as adipocyte hypertrophy, fibrosis, immune cell recruitment, are nevertheless present in the thighs of lipedema patients, which are typically associated with increased HOMA-IR in primarily obese collectives (22).

Our present study has some limitations that should be considered. In view of the high inter-individual variability of human SAT samples, the relatively small total number of cases and control subjects requires careful interpretation of the results. Moreover, without an objective biomarker, the diagnosis of lipedema remains subject to a certain bias and relies on the presence of extensive expertise in the diagnosis of the disease. In this study, independent diagnosis validation and the patient’s interest in surgical symptom reduction with liposuction reduced this factor to a large extent. Another limitation might be the location of adipose tissue biopsy harvesting: The human SAT consists of two distinct compartments separated by Scarpa’s fascia into superficial and deep SAT. It was recently demonstrated that these depots are functionally and morphologically different in terms of adipocyte size and inflammatory gene expression levels (56). Since it is not possible to differentiate between the two depots when using the small liposuction incisions in the harvesting procedure, samples were always taken *en bloc* from both regions. Unlike in punching procedures, the surgical dissection ensures that sufficient amount of tissue is obtained from both compartments. This *en bloc* harvesting technique provides comparability between all samples and individuals, regardless of local tissue variances, but does not allow differentiation between the two layers.

## 5 Conclusions

Lipedema SAT is associated with progressive interstitial fibrosis, inflammatory processes and an elevated proportion of M2-like macrophages in early stages of the disease that is followed by adipocyte hypertrophy and a more pro-inflammatory profile in later stages. The observed SAT changes are mainly limited to the clinically affected areas and provide objective measure to assist diagnosis, but are also present in other adipose regions to a more limited extent. The characteristics of the inflammatory response, and in particular the increased presence of anti-inflammatory, M2-like macrophages clearly distinguishes lipedema from primary obesity and may reflect a key role in the pathophysiology and symptoms of lipedema at different stages. Thus, the regulation of M2-like macrophages warrants further studies in relation to the clinical characterization of patients and development of diagnostic tools of the disease. Although altered lipid metabolism is closely related to the inflammatory response of adipose tissue in obesity, key predictors of metabolic risk were not altered in the lipedema cohort studied. In contrast, some parameters of metabolic health were improved in lipedema patients compared to BMI- and age-matched individuals. We conclude that a better understanding of the distinct composition of plasma lipids in conjunction with an assessment of inflammatory processes may help to better characterize the underlying patho-mechanisms of lipedema.

## Data availability statement

The original contributions presented in the study are included in the article/**Supplementary Material**. Further inquiries can be directed to the corresponding author.

## Author contributions

Conceptualization, TS, MG, PK, SG and FG-C. Methodology, TS, MG, PK, SG, GS and FG-C. Formal analysis, PK, SG, KL, and FG-C. Investigation, PK, SG, KL, and FG-C. Resources, TS and MG. Data curation, PK, SG, KL, and FG-C. Writing—original draft preparation, PK and SG. Writing—review and editing, TS, MG, SG and FG-C. Visualization, PK and SG. Supervision, TS and MG. Project administration, TS and MG. All authors have read and agreed to the published version of the manuscript.

## Supplementary material

Supplementary Figure 1 Stage dependent increase of adipocyte hypertrophy in thigh adipose tissue of lipedema patients. **(A)** Representative images of hematoxylin and eosin (H&E) stained adipose tissue sections from the abdominal and thigh regions of lipedema patients from stage I to stage III (100x magnification; scale bar 100 µm).

Supplementary Figure 2 Stage dependent increase of interstitial fibrosis occurs in affected extremities of lipedema patients. **(A, B)** Representative images of Sirius red-stained interstitial fibrosis in abdomen **(A)** and thigh **(B)** SAT sections of lipedema patients from stage I to stage III (200x magnification; scale bar 100 µm).

Supplementary Figure 3 Marker expression in abdominal biopsies of lipedema patients compared to controls. **(A)** Gene expression analysis of marker genes related to adipogenesis, inflammation, fibrosis and mitochondrial function in abdomen **(A)** of lipedema patients (n=30, white bars) depicted as relative gene expression of all non-lipedema control subjects (n=14). **(B–D)** Gene expression analysis of marker genes related to adipogenesis, inflammation, fibrosis and mitochondrial function in abdomen SAT of lipedema patients stage I (n=9, white bars, **B**), stage II (n=16; grey bars, **C**) and stage III (n=7; grey bars, **D**) depicted as fold change of all non-lipedema control subjects (n=14). All data are represented as mean ± SEM. *p<0.05; **p<0.005 using unpaired, two-tailed t-test with Welch correction.

Supplementary Figure 4 Analysis of marker genes for angiogenesis in thigh lipedema patients compared to controls. (**A**) Gene expression analysis of marker genes related to angiogenesis in abdomen and thigh biopsies of lipedema patients (n=30; white bars) depicted as relative gene expression of all non-lipedema control subjects (n=14).). **(B–D)** Gene expression analysis of marker genes related to angiogenesis in abdomen SAT and thigh SAT of lipedema patients stage I (n=9, white bars, **B**), stage II (n=16; grey bars, **C**) and stage III (n=7; grey bars, **D**) depicted as relative gene expression of all non-lipedema control subjects (n=14). All data are represented as mean ± SEM. *p<0.05; using unpaired, two-tailed t-test with Welch correction.

Supplementary Figure 5 Pro-inflammatory macrophages staining. **(A, B)** Representative bright field analysis (BF) and bright field merged with immunofluorescent signals (BF MERGE) of macrophage infiltration in thigh adipose tissue of lipedema patients. Green arrows highlight CD68+ pan-macrophage and red arrows mark CD86+ pro-inflammatory macrophage **(A)** or CD206+ anti-inflammatory macrophages **(B)**. **(C, D)** Representative images of immunofluorescence staining of macrophages in lipedema patient to detect DAPI nuclei (blue), CD68+ pan-macrophage (green arrows) and CD86+ pro-inflammatory macrophage (red arrows) markers **(C)** or CD206+ anti-inflammatory macrophages **(D)**. Merged: yellow spots indicate co-staining of anti-CD68 and anti-CD86 antibodies). (100x magnification; scale bar 100 µm).
