# Supplementary figures and images for: Lipedema stage affects adipocyte hypertrophy, subcutaneous adipose tissue inflammation and interstitial fibrosis
Source: Front Immunol. 2023 Jul 28;14:1223264. doi: 10.3389/fimmu.2023.1223264 (PMC10417720; doi:10.3389/fimmu.2023.1223264)

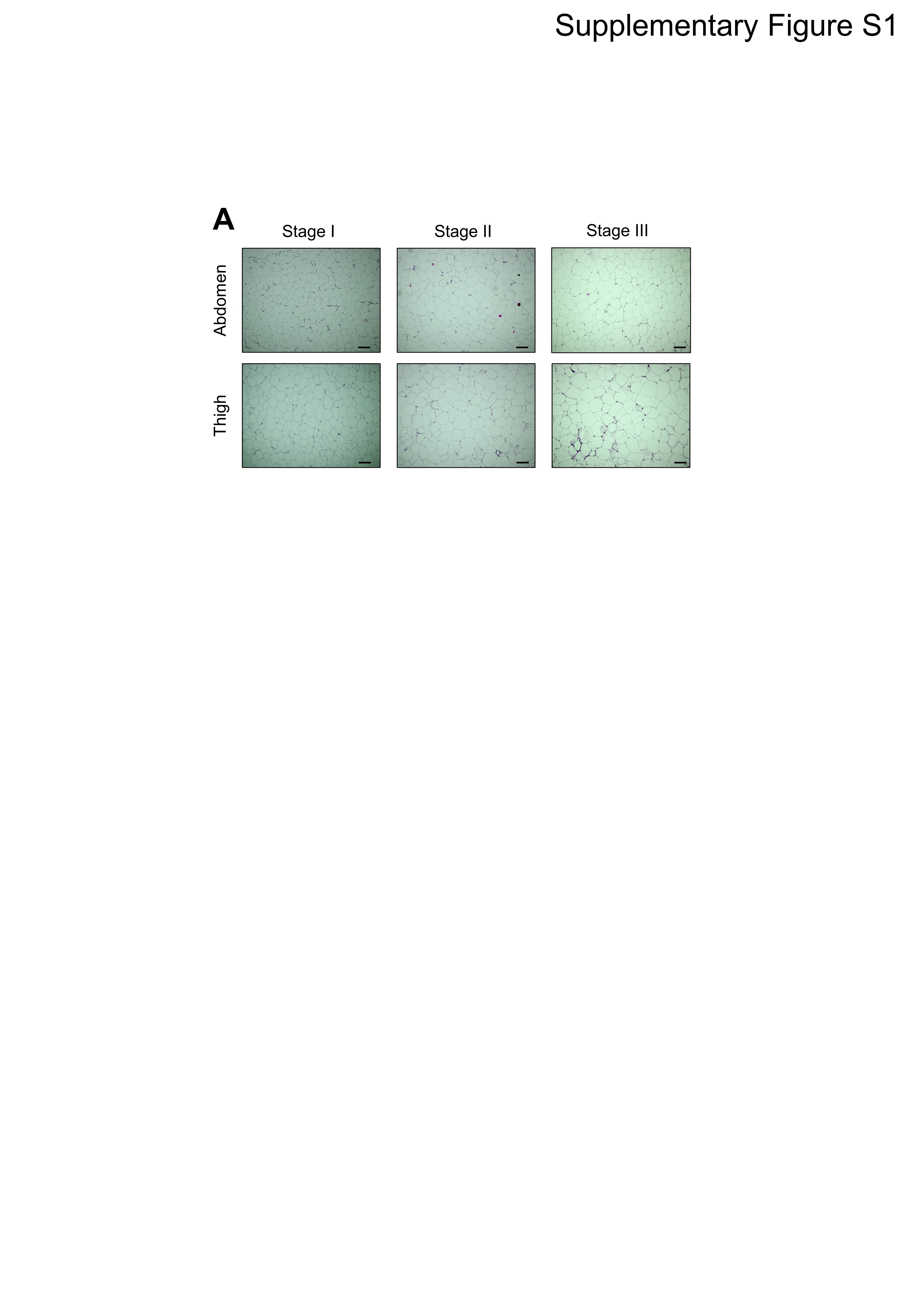

Supplement: Supplementary Figure 1 — Stage dependent increase of adipocyte hypertrophy in thigh adipose tissue of lipedema patients. (A) Representative images of hematoxylin and eosin (H&E) stained adipose tissue sections from the abdominal and thigh regions of lipedema patients from stage I to stage III (100x magnification; scale bar 100 µm). [file Image_1.tif]

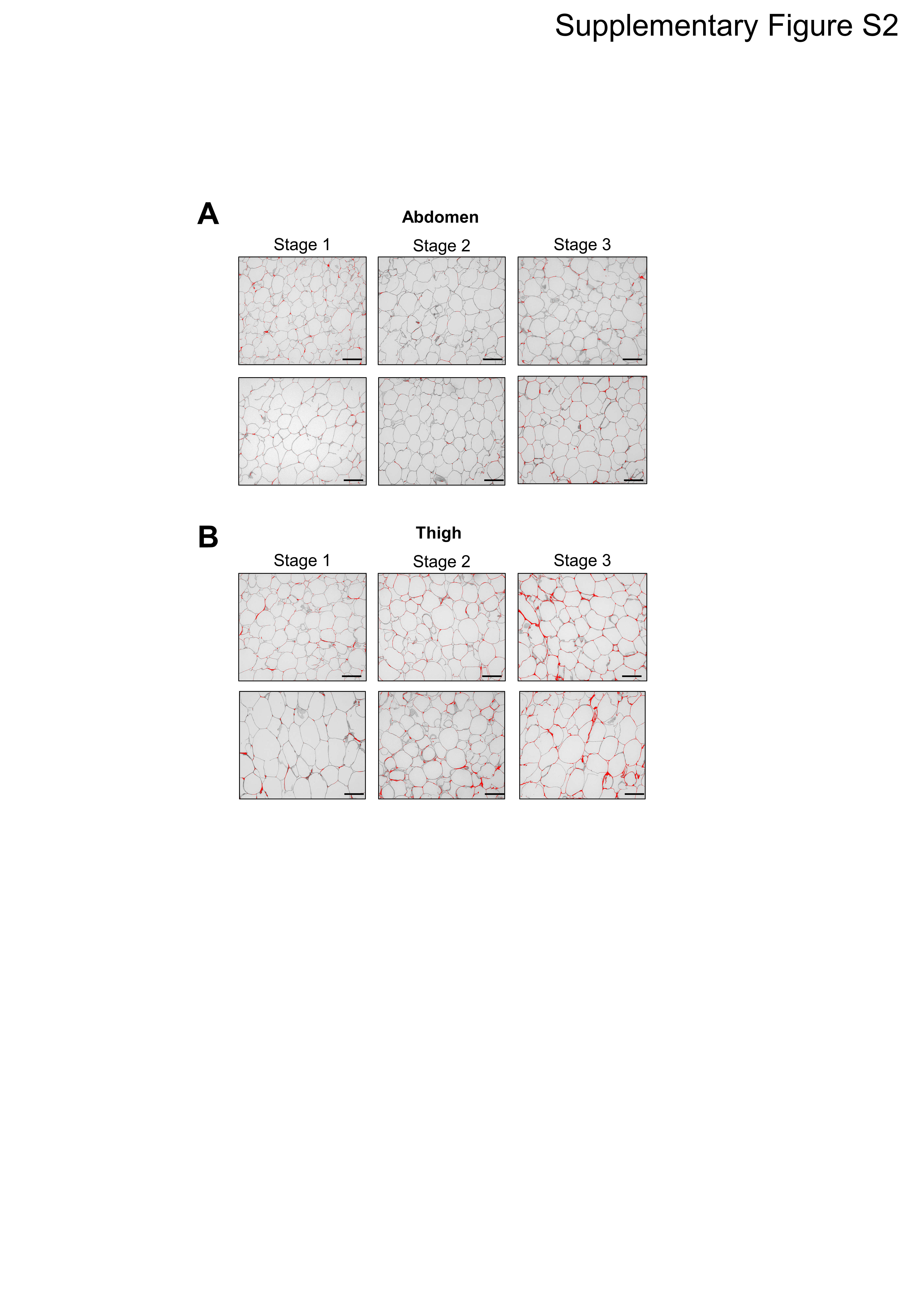

Supplement: Supplementary Figure 2 — Stage dependent increase of interstitial fibrosis occurs in affected extremities of lipedema patients. (A, B) Representative images of Sirius red-stained interstitial fibrosis in abdomen (A) and thigh (B) SAT sections of lipedema patients from stage I to stage III (200x magnification; scale bar 100 µm). [file Image_2.tif]

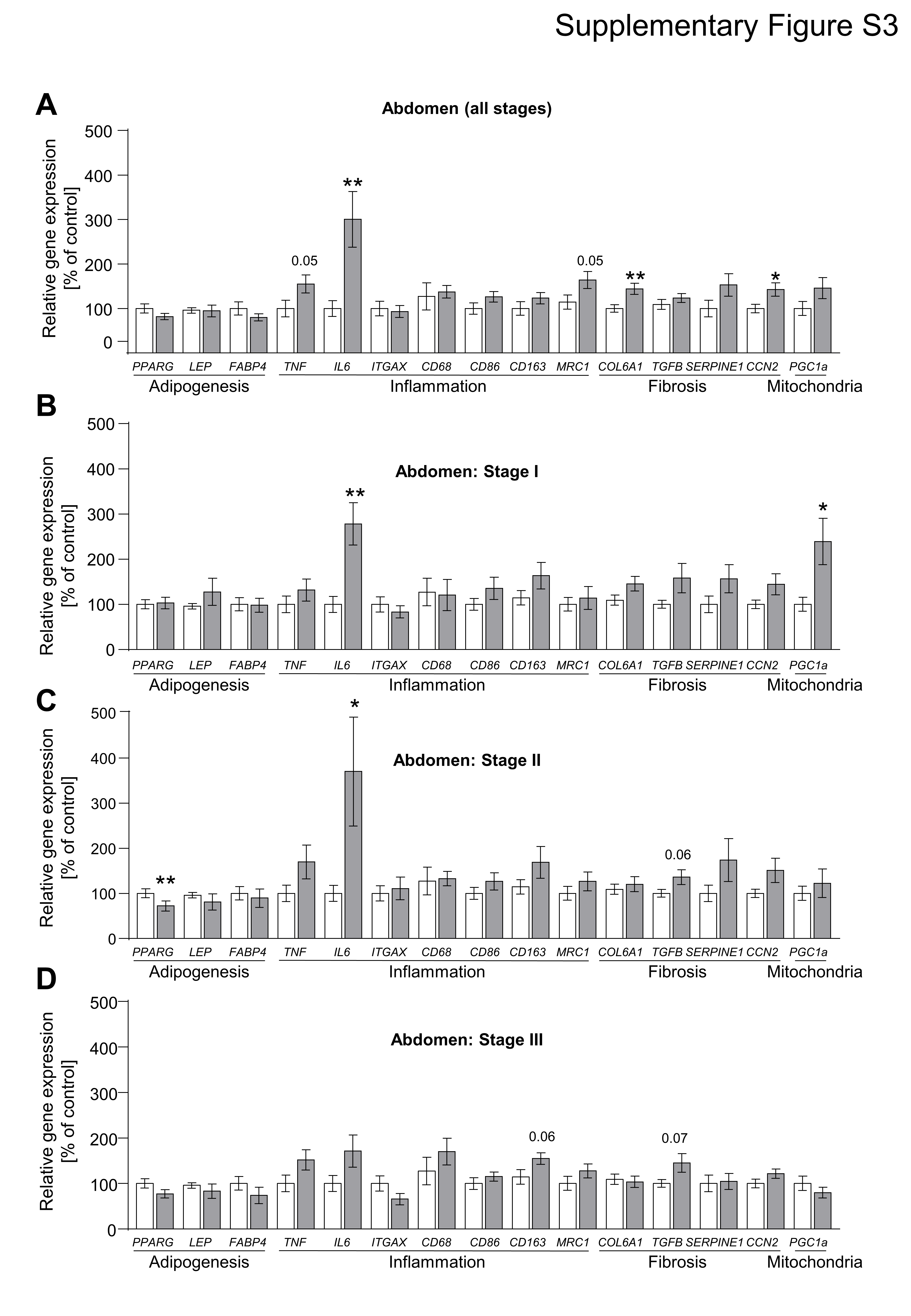

Supplement: Supplementary Figure 3 — Marker expression in abdominal biopsies of lipedema patients compared to controls. (A) Gene expression analysis of marker genes related to adipogenesis, inflammation, fibrosis and mitochondrial function in abdomen (A) of lipedema patients (n=30, white bars) depicted as relative gene expression of all non-lipedema control subjects (n=14). (B–D) Gene expression analysis of marker genes related to adipogenesis, inflammation, fibrosis and mitochondrial function in abdomen SAT of lipedema patients stage I (n=9, white bars, B), stage II (n=16; grey bars, C) and stage III (n=7; grey bars, D) depicted as fold change of all non-lipedema control subjects (n=14). All data are represented as mean ± SEM. *p<0.05; **p<0.005 using unpaired, two-tailed t-test with Welch correction. [file Image_3.tif]

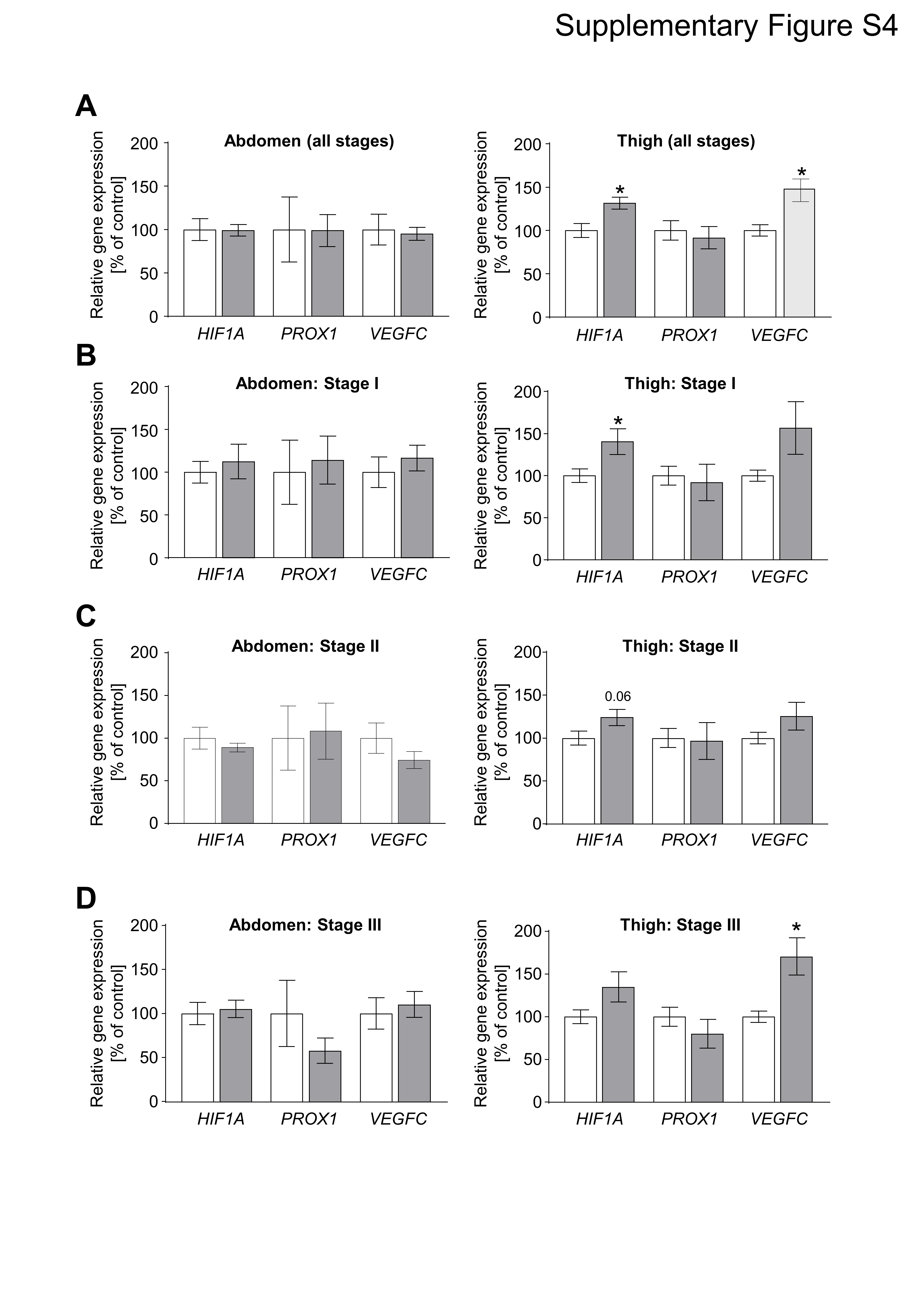

Supplement: Supplementary Figure 4 — Analysis of marker genes for angiogenesis in thigh lipedema patients compared to controls. (A) Gene expression analysis of marker genes related to angiogenesis in abdomen and thigh biopsies of lipedema patients (n=30; white bars) depicted as relative gene expression of all non-lipedema control subjects (n=14).). (B–D) Gene expression analysis of marker genes related to angiogenesis in abdomen SAT and thigh SAT of lipedema patients stage I (n=9, white bars, B), stage II (n=16; grey bars, C) and stage III (n=7; grey bars, D) depicted as relative gene expression of all non-lipedema control subjects (n=14). All data are represented as mean ± SEM. *p<0.05; using unpaired, two-tailed t-test with Welch correction. [file Image_4.tif]

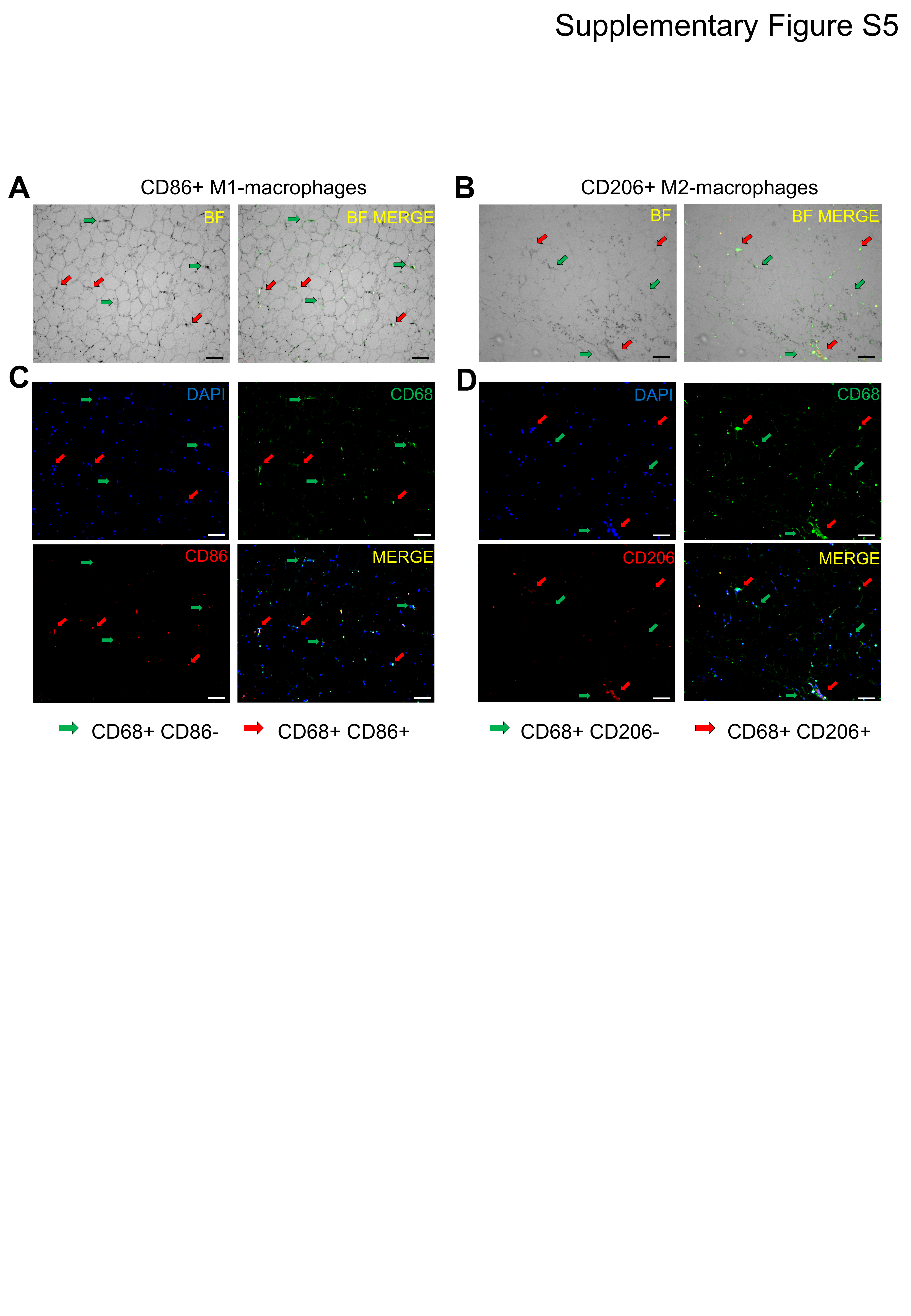

Supplement: Supplementary Figure 5 — Pro-inflammatory macrophages staining. (A, B) Representative bright field analysis (BF) and bright field merged with immunofluorescent signals (BF MERGE) of macrophage infiltration in thigh adipose tissue of lipedema patients. Green arrows highlight CD68+ pan-macrophage and red arrows mark CD86+ pro-inflammatory macrophage (A) or CD206+ anti-inflammatory macrophages (B). (C, D) Representative images of immunofluorescence staining of macrophages in lipedema patient to detect DAPI nuclei (blue), CD68+ pan-macrophage (green arrows) and CD86+ pro-inflammatory macrophage (red arrows) markers (C) or CD206+ anti-inflammatory macrophages (D). Merged: yellow spots indicate co-staining of anti-CD68 and anti-CD86 antibodies). (100x magnification; scale bar 100 µm). [file Image_5.tif]
