# Supplementary material for: Lipedema stage affects adipocyte hypertrophy, subcutaneous adipose tissue inflammation and interstitial fibrosis
Source: Front Immunol. 2023 Jul 28;14:1223264. doi: 10.3389/fimmu.2023.1223264 (PMC10417720; doi:10.3389/fimmu.2023.1223264)
Supplement: Supplementary file 6 [file Table_1.docx]

Supplementary Material

Lipedema stage affects adipocyte hypertrophy, subcutaneous adipose tissue inflammation and interstitial fibrosis

Philipp Kruppa^1,2,3,*,†^; Sabrina Gohlke^2,†^; Kamila Lapinski^1,2,3^; Francisco Garcia-Carrizo^2,4^; George Soultoukis^2^; Manfred Infanger^3^; Tim J. Schulz^2,4,5^; Mojtaba Ghods^1^

^1^Department of Plastic, Aesthetic and Reconstructive Microsurgery/Hand surgery, Hospital Ernst von Bergmann, Potsdam, Germany

^2^Department of Adipocyte Development and Nutrition, German Institute of Human Nutrition (DIfE) Potsdam-Rehbrücke, Nuthetal, Germany

^3^Otto-von-Guericke University Magdeburg, Department of Plastic, Aesthetic and Hand Surgery, Magdeburg, Germany

^4^German Center for Diabetes Research (DZD), München, Neuherberg, Germany

^5^Institute of Nutritional Science, University of Potsdam, Potsdam-Rehbrücke, Nuthetal, Germany

†These authors contributed equally to this work and share first authorship.

*** Correspondence:**

Philipp Kruppa, M.D.
Department of Plastic, Aesthetic and Reconstructive Microsurgery/Hand surgery
Hospital Ernst von Bergmann
Charlottenstraße 72
14467 Potsdam, Germany
Phone: +49 331 2413 7802
E-mail: [kruppaph@gmail.com](mailto:kruppaph@gmail.com)

# Table S1: Quantitative real-time PCR primer sequences.

| **Gene** | **Sequence (5´to 3´)** |
| --- | --- |
| ***ACTB*** | Fw: ATGATGAGAGGCAGCAAGATGG |
|  | Rev: GCTGATCCACATCTGCTGGAA |
| ***CD11C*** | Fw: TGACATTGCATCGAAGCCCTC |
|  | Rev: TCCGTACCCTCAATGGCAAAG |
| ***CD68*** | Fw: GCTACATGGCGGTGGAGTACAA |
|  | Rev: ATGATGAGAGGCAGCAAGATGG |
| ***CD86*** | Fw: TAGGTCACAGCAGAAGCAGC |
|  | Rev: AATCAAAACTTGTGCGGCCC |
| ***CD163*** | Fw: GCTACATGGCGGTGGAGACAA |
|  | Rev: ATGATGAGAGGCAGCAAGATGG |
| ***CD206*** | Fw: GGGTTGCTATCACTCTCTATGC |
|  | Rev: TTTCTTGTCTGTTGCCGTAGTT |
| ***ColVIa1*** | Fw: ACTCAGAGGGACACCAGACC |
|  | Rev: GAGCCTGGGATGAAGTCAAA |
| ***CTGF*** | Fw: CAGCATGGACGTTCGTCTG |
|  | Rev: AACCACGGTTTGGTCCTTGG |
| ***FABP4*** | Fw: CATAAAGAGAAAACGAGAGGATGATAAA |
|  | Rev: CCCTTGGCTTATGCTCTCTCA |
| ***IL6*** | Fw: ACTCACCTCTTCAGAACGAATTG |
|  | Rev: CCATCTTTGGAAGGTTCAGGTTG |
| ***Leptin*** | Fw: GGCTTTGGCCCTATCTTTTC |
|  | Rev: CCAAACCGGTGACTTTCTGT |
| ***PAI1*** | Fw: GCTTGGTGATGTCTGGTCCAT |
|  | Rev: CACCACTTGTTGCTCCATATCCT |
| ***PGC1a*** | Fw: GGTCTCTCCTTGCAGCACAAG |
|  | Rev: CTGGGATGACCGAAGTGCTT |
| ***PPARg*** | Fw: GACCACTCCCACTCCTTTGA |
|  | Rev: GATGCAGGCTCCACTTTGAT |
| ***TGFb*** | Fw: ACGTGGAGCTGTACCAGAAATA |
|  | Rev: GGCGAAAGCCCTCAATTTCC |
| ***TNFa*** | Fw: TGGAGCTGGCCGAGGAG |
|  | Rev: AGCAGGCAGAAGAGCGTGG |
| ***HIF1A*** | Fw: ATCCATGTGACCATGAGGAAATG |
|  | Rev: TCGGCTAGTTAGGGTACACTTC |
| ***PROX1*** | Fw: AGGTTGGTGGAGTGATGGAG |
|  | Rev: ATGCAGATGCTGTCCCTACC |
| ***VEGFC*** | Fw: GGCTGGCAACATAACAGAGAA |
|  | Rev: CCCCACATCTATACACACCTCC |
